# Hyperbaric oxygen therapy alters bowel perfusion and improves outcomes in patients with treatment-refractory ulcerative colitis: a prospective pilot trial

**DOI:** 10.1093/ecco-jcc/jjag065

**Published:** 2026-05-14

**Authors:** Lieven Mulders, Maarten Pruijt, Joep Van Oostrom, Esmerij Van Der Zanden, Andra Neefjes-Borst, Pim Koelink, Manon Wildenberg, Wouter De Jonge, Milan Ridderikhof, Rob Van Hulst, Geert D’Haens, Krisztina Gecse

**Affiliations:** Department of Gastroenterology and Hepatology, Amsterdam UMC, University of Amsterdam, Amsterdam Gastroenterology Endocrinology Metabolism, Amsterdam, The Netherlands; Department of Gastroenterology and Hepatology, Amsterdam UMC, University of Amsterdam, Amsterdam Gastroenterology Endocrinology Metabolism, Amsterdam, The Netherlands; Department of Gastroenterology and Hepatology, Amsterdam UMC, University of Amsterdam, Amsterdam Gastroenterology Endocrinology Metabolism, Amsterdam, The Netherlands; Department of Gastroenterology and Hepatology, Amstelland Ziekenhuis, Amstelveen, The Netherlands; Department of Pathology, Amsterdam UMC, Vrije Universiteit Amsterdam, Amsterdam Gastroenterology Endocrinology Metabolism, Amsterdam, The Netherlands; Tytgat Institute for Liver and Intestinal Research, Amsterdam UMC, University of Amsterdam, Amsterdam Gastroenterology Endocrinology Metabolism, Amsterdam, The Netherlands; Tytgat Institute for Liver and Intestinal Research, Amsterdam UMC, University of Amsterdam, Amsterdam Gastroenterology Endocrinology Metabolism, Amsterdam, The Netherlands; Tytgat Institute for Liver and Intestinal Research, Amsterdam UMC, University of Amsterdam, Amsterdam Gastroenterology Endocrinology Metabolism, Amsterdam, The Netherlands; Department of Hyperbaric Medicine, Amsterdam UMC, University of Amsterdam, Amsterdam, The Netherlands; Department of Emergency Medicine, Amsterdam UMC, University of Amsterdam, Amsterdam, The Netherlands; Department of Hyperbaric Medicine, Amsterdam UMC, University of Amsterdam, Amsterdam, The Netherlands; Department of Anesthesiology, Amsterdam UMC, University of Amsterdam, Amsterdam, The Netherlands; Department of Gastroenterology and Hepatology, Amsterdam UMC, University of Amsterdam, Amsterdam Gastroenterology Endocrinology Metabolism, Amsterdam, The Netherlands; Department of Gastroenterology and Hepatology, Amsterdam UMC, University of Amsterdam, Amsterdam Gastroenterology Endocrinology Metabolism, Amsterdam, The Netherlands

**Keywords:** ulcerative colitis, hyperbaric oxygen therapy, neovascularization

## Abstract

**Background:**

Hyperbaric oxygen therapy (HBOT) delivers 100% oxygen in a pressurized chamber and enhances tissue oxygenation and neovascularization. While effective in radiation proctitis, acute severe ulcerative colitis, and perianal fistulizing Crohn’s disease, its role in refractory ulcerative colitis (UC) remains underexplored.

**Methods:**

PARADOX was a prospective, open-label, phase 2a pilot trial in biologic-experienced patients with moderate-to-severe refractory UC (total Mayo > 5, Mayo endoscopic subscore (MES) ≥ 2, failure of ≥ 2 advanced therapies). Patients received 10 or 20 daily HBOT sessions (2.4 atmospheres absolute, 120 minutes/day) while continuing stable background therapy. The primary endpoint was composite clinical and endoscopic response at week 12 (≥ 3-point and 30% Mayo score reduction, rectal bleeding score = 0, and ≥ 1-point decrease in MES). Secondary outcomes included symptomatic, biochemical, and transmural response by intestinal ultrasound (IUS), including perfusion metrics via contrast-enhanced ultrasound (CEUS).

**Results:**

In total, 16 patients (8 per group) were included in this pilot study. At week 12, composite response was achieved in 2/8 (10-session) and 4/8 (20-session) patients. Improvements in clinical, endoscopic, and IUS parameters were observed in both groups. Clinical responders showed increased CEUS perfusion at week 12 (peak enhancement Δ12.1 dB), while non-responders declined (Δ –5.4 dB). This pattern was consistent across wash-in/wash-out metrics, supporting a volume perfusion-based response. HBOT was well tolerated, with no serious adverse events or treatment discontinuations.

**Conclusion:**

HBOT is a well-tolerated adjunctive therapy in refractory UC, which improved clinical outcomes and perfusion kinetics. These findings support further evaluation in a randomized, dose-optimized trial.

Euclinicaltrials.eu, EU CT number 2024–515278-28-00

## Graphical Abstract

**Graphical Abstract:**
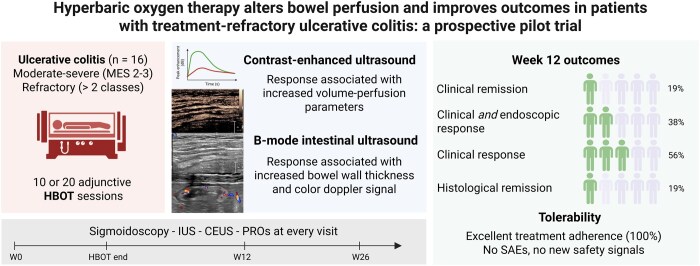

## 1. Introduction

Ulcerative colitis (UC) is a chronic inflammatory bowel disease characterized by relapsing episodes of urgency, bloody diarrhea, abdominal pain, and fatigue, profoundly impacting patient quality of life. Despite the expansion of available therapies, a significant subset of patients remains refractory to multiple medical treatments, often necessitating proctocolectomy.^1–3^ Thus, identifying novel adjunctive treatments remains a clinical priority.

Hyperbaric oxygen therapy (HBOT) is a treatment which involves inhalation of 100% oxygen under increased atmospheric pressure, promotes tissue oxygenation and stimulates neovascularization, making it an attractive therapeutic candidate for ischemia-driven inflammatory diseases.^4^ HBOT is effective in chronic non-healing wounds and radiation-induced proctitis and enteritis, and early reports in UC have suggested high response rates (83%, *n* = 425) with generally mild and reversible adverse events.^5–8^ A cost-effectiveness analysis further suggested that HBOT may reduce hospitalization duration, rescue therapies, and emergency colectomy in patients with acute severe UC.^5^^,^^9^ Similar benefit has been reported in other refractory IBD phenotypes, including perianal fistulizing Crohn’s disease.^10^^,^^11^ However, existing evidence is limited by heterogeneous protocols, absence of placebo-controlled data, and uncertainty about optimal dosing regimens.^7^

Mechanistically, HBOT likely exerts therapeutic effects by modulating mucosal hypoxia.^12^ Under physiological conditions, the intestinal mucosa maintains a steep oxygen gradient essential for barrier integrity.^13^ During active inflammation, increased oxygen consumption and leukocyte infiltration exacerbate mucosal hypoxia.^14^ Hypoxia-inducible factors (HIFs), particularly HIF-1α, are key regulators responding to hypoxic stress, orchestrating anti-inflammatory responses, promoting epithelial barrier function (eg, through tight junction protein claudin-1), and mucosal repair.^15^^,^^16^ HBOT exploits the “hyperoxic-hypoxic paradox,” where transient hyperoxia during treatment improves perfusion and reduces edema, followed by relative hypoxia in between sessions.^17^^,^^18^ This paradoxical stimulus may elevate mucosal HIF-1α levels and initiate regenerative and anti-inflammatory cascades. Preclinical studies support these mechanisms, demonstrating HBOT-induced increases in mucosal HIF-1α associated with histological improvement.^19–21^ Furthermore, emerging data also suggests HBOT may influence intestinal stem cell activation, gut microbiota composition, and drug delivery through enhanced perfusion.^20^^,^^22^

By improving tissue oxygenation, perfusion and promoting mucosal repair, we hypothesized that in biologic-refractory UC, HBOT could re-open a therapeutic window for disease stabilization and avoidance of colectomy. PARADOX evaluated the preliminary efficacy of different HBOT dosing regimens, with mechanistic assessment focused on vascular conditioning. In addition to clinical, endoscopic, histologic, and patient-reported outcomes, we incorporated intestinal ultrasound (IUS) and contrast-enhanced ultrasound (CEUS) as translational imaging tools to quantify changes in bowel wall vascularization over time, providing a non-invasive readout of HBOT’s hypothesized mechanism.^23^^,^^24^ Finally, safety, tolerability and feasibility were assessed in this high-risk patient population.

## 2. Methods

### Design

PARADOX was an investigator-initiated, non-randomized, open-label, phase 2a dose-ranging pilot trial carried out at Amsterdam UMC, a tertiary IBD referral center in Amsterdam, The Netherlands, in collaboration with regional hyperbaric oxygen facilities. Patients were enrolled from January 2023 through December 2024.

The PARADOX study was conducted in accordance with the Declaration of Helsinki (2013 revision), Good Clinical Practice guidelines, and CONSORT recommendations. The study received IRB approval from the Medical Ethical Committee of Amsterdam UMC (METC Amsterdam UMC: 2022.0158) and was prospectively registered in the EU Clinical Trials Register: 2024–515278-28-00.

### Participants

Patients enrolled in PARADOX were aged ≥ 16 years, with a diagnosis of UC of at least 4 months confirmed by clinical evaluation supported by endoscopic and histopathologic criteria.^25^ Inclusion criteria required moderate-to-severe disease activity, defined by a total Mayo score of ≥ 5 *and* a Mayo endoscopic subscore (MES) of ≥ 2.^26^ Patients previously failed at least two classes of advanced medical treatments, including biologics or small molecules, and maintained optimized and stable doses of background medications for at least 8 weeks prior to enrollment and throughout the study. Initiation of corticosteroids during treatment or follow-up was considered treatment failure. All participants provided written informed consent prior to enrollment.

### Study procedures

**Figure 1.**
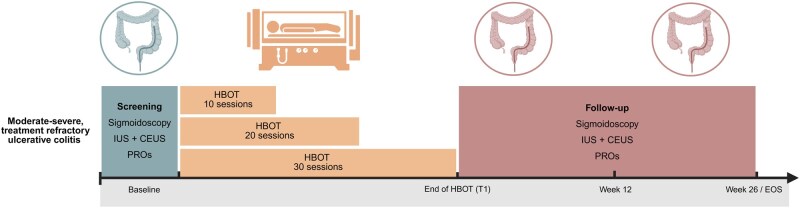
PARADOX trial design. Schematic overview of the PARADOX trial on hyperbaric oxygen therapy (HBOT) in patients with moderate-to-severe (total Mayo score [TMS] ≥ 5 and Mayo endoscopic subscore [MES] 2-3), treatment-refractory (failure of ≥ 2 advanced therapy classes) ulcerative colitis. Sequential cohorts were treated with 10 sessions (2 weeks), 20 sessions (4 weeks), or 30 sessions (6 weeks) of HBOT, depending on interim analyses of predefined feasibility criteria at week 12. Baseline and follow-up assessments included sigmoidoscopy with biopsies, intestinal ultrasound (IUS) with contrast-enhanced ultrasound (CEUS), and patient-reported outcomes (PROs). Follow-up assessments were anchored to the final HBOT session and performed at the end of HBOT (T1), week 12, and week 26. Created with BioRender.com.

Patients were sequentially enrolled into 3 treatment groups of 8 patients, receiving either 10, 20, or 30 HBOT sessions during 2, 4, or 6 weeks, respectively, for optimal dose finding (Figure 1). If a cohort reached an a priori defined response rate of at least 50%, no further dose escalation in subsequent cohorts was carried out. Response was defined as a composite clinical and endoscopic response by a reduction of ≥ 3 points and 30% from baseline in the total Mayo score, a rectal bleeding subscore (RB) of 0, and a ≥ 1-point decrease in MES at week 12.

HBOT was administered using supervised, multiplace hyperbaric chambers. Patients were compressed from 1 atmosphere absolute (ATA; sea level) to a treatment pressure of 2.4 ATA while breathing 100% oxygen, including 2 intermittent 5-minute air breaks to reduce the potential risk of oxygen-induced central nerve system toxicity. Each session lasted 120 minutes and was scheduled on consecutive weekdays (ie, Mon-Fri) and suspended during weekends and national holidays.

During the 26-week follow-up, patients went through sigmoidoscopy with biopsies and intestinal ultrasound (IUS) with IV contrast (CEUS) at baseline (T0), at the end of HBOT (T1, within 3 days of last HBOT session), and W12 (T2; Figure 1).

All CEUS examinations were performed by one sonographer (MP, > 3 years of experience) using an Epiq 5G Philips machine with the L12-5 MHz transducer with contrast specific pre-sets.^23^ All CEUS measurements were performed in the sigmoid colon at all timepoints, even when the bowel wall thickness (BWT) was normal. The focus region was set just below the area of interest and the gain was kept constant during the study. 2.4 mL of contrast agent (SonoVue, Bracco) followed by 10 mL of 0.9% saline was intravenously administered via an antecubital vein catheter. Directly after administration, a cine-loop was recorded for 90 seconds. This procedure was performed twice in the same bowel segment at every visit, after which the cine-loop with the best image quality was selected for analysis.

A single sonographer (MP) analyzed all CEUS cine-loops using VueBox (Bracco). The entire bowel wall, together with the adjacent mesentery, was delineated using the peritoneum as the boundary. Motion compensation was subsequently performed using the peak enhancement (PE) frame as the reference. Images displaying more than 1 cm of displacement beyond the delineated area were excluded. The cine-loop with the best image quality and the fewest excluded frames was selected for quantitative analysis. Three regions of interest (ROIs) were subsequently defined: ROI1 included the entire anterior bowel wall; ROI2 included only the submucosa; and ROI3 comprised a single vessel located within either the mucosa or submucosa. CEUS parameters were extracted for each ROI and expressed both as linear values and log-converted data (decibel [dB] or seconds [s]). Additional details are provided in the Supplementary Methods.

### Outcomes

The primary efficacy outcome was the composite clinical and endoscopic response at W12, defined as a reduction of ≥ 3 points and 30% from baseline in the total Mayo score, a rectal bleeding subscore (RB) of 0, and a ≥ 1-point decrease in MES. All endoscopic examinations were centrally reviewed and adjudicated according to the validated VISA 2 + 1 algorithm.^27^ Secondary efficacy outcomes included clinical remission and response, endoscopic improvement, histologic remission and improvement, normalization of biochemical parameters, and patient-reported outcomes at T1 and W12. Methodological details are provided in Supplementary Methods.

Exploratory mechanistic outcomes focused on imaging biomarkers of vascular conditioning. IUS response was defined as a decrease in BWT of > 25%, or ≥ 2 mm, or 1 mm and one point reduction in color doppler signal (CDS) in the sigmoid. IUS remission was defined as a BWT of ≤ 3 mm and a CDS of 0 in the sigmoid. CEUS perfusion parameters included time-to-peak, peak enhancement (PE), wash-in rate (WiR), wash-out rate (WoR), wash-in and wash-out area under the curve (WiWoAUC), rise time, and fall time. Safety and tolerability were assessed via the frequency and proportion of treatment-emergent AEs (TEAEs), treatment-emergent SAEs (TESAEs), and TEAEs leading to HBOT discontinuation. Identified AEs were graded according to the Common Terminology Criteria for Adverse Events Version 5 with attribution by the investigator.

### Statistical analysis

All analyses were conducted in the intention-to-treat (ITT) population, defined as patients who received ≥ 1 HBOT session. Binary efficacy outcomes at W12 were summarized as proportions with Wilson 95% CI. The primary analysis applied non-responder imputation for missing data.

Continuous efficacy outcomes were summarized as observed means (SD) at each timepoint and as baseline-anchored mean changes with 95% CIs. No formal between-regimen hypothesis testing was planned, regimen-stratified summaries are descriptive.

Mechanistic outcomes were analyzed separately. For CEUS, analyses were restricted to the total bowel wall ROI, and sensitivity analyses were applied using a quality-of-fit threshold of at least 70%. Observed mean values and baseline-anchored changes were summarized overall and stratified by W12 composite response and HBOT regimen. Exploratory responder analyses examined associations between early perfusion changes (ΔPE, ΔWiWoAUC at T1) and W12 composite response using ROC analyses. B-mode ultrasound parameters were compared descriptively overall and between responders and non-responders using *t*-tests or Fisher’s exact test, as appropriate.

Safety analyses were conducted on the ITT population. Adverse events were summarized as counts and proportions, with exposure-adjusted incidence rates per patient year (PY) and for HBOT-related TEAEs per 100 HBOT sessions.

A *P*-value < .05 was considered statistically significant. However, all findings were interpreted with caution given the exploratory nature and limited sample size of the study. No adjustments for multiple comparisons were applied.

## 3. Results

Between January 2023 and December 2024, 17 patients were screened for eligibility in the PARADOX study, of whom 16 (94%) were enrolled (Figure S1). Baseline characteristics reflected a refractory patient population (Table 1): across both groups, 13 of 16 patients (81%) had previously failed 3 or more advanced therapies and 9 of 16 patients (56%) presented with a MES of 3. While overall characteristics were broadly comparable between the 2 cohorts, baseline disease activity (as indicated by the UCEIS, MES 3 and fecal calprotectin) tended to be numerically higher in the 10-session cohort, whereas in the 20-session cohort more patients had prior exposure to at least 2 advanced therapies (Table 1).

**Table 1.** Baseline demographics and disease characteristics.

	10 sessions HBOT *N* = 8	20 sessions HBOT *N* = 8
**Demographics and treatment history**		
**Age in years, median [IQR]**	48 [45-55]	64 [45-77]
**Sex, female, *n* (%)**	5 (63)	4 (50)
**Disease extent (Montreal classification), *n* (%)**		
** Rectum (E1)**	0	1 (13)
** Left-sided colitis (E2)**	4 (50)	2 (25)
** Pancolitis (E3)**	4 (50)	5 (63)
**Disease duration in years, median [IQR]**	12 [10-16]	9 [6-14]
**No. of unique prior advanced therapies, *n* (%)**		
** 2**	2 (25)	1 (13)
** 3**	3 (38)	4 (50)
** 4**	1 (13)	2 (25)
** 5**	2 (25)	1 (13)
**Concomitant medications^a^, *n* (%)**		
** Mesalazine**	4 (50)	3 (38)
** Corticosteroids**	3 (38)	3 (38)
** Adalimumab**	0	1 (13)
** Ustekinumab**	1 (13)	5 (63)
** Vedolizumab**	0	1 (13)
** Upadacitinib**	1 (13)	1 (13)
** Tofacitinib**	3 (38)	2 (25)
** Filgotinib**	3 (38)	0
**Combination treatment of advanced medical therapies, *n* (%)**	0	3 (38)
**Prior appendectomy, *n* (%)**	2 (25)	3 (38)
**Disease characteristics**		
**Mayo score, median [IQR]**	10.5 [6.8-11]	9 [7-9.3]
**MES 3, *n* (%)**	6 (75)	3 (38)
**UCEIS, median [IQR]**	6.5 [4.8-7.0]	4.0 [2.8-5.0]
**SCCAI, median [IQR]**	10 [6-11]	7 [5-8]
**hs-CRP (mg/L), median [IQR]**	4.2 [1.2–9.3]	1.8 [0.8–2.3]
**Fecal calprotectin (µg/g), median [IQR]**	1127 [760-3253]	543 [208-763]
**Bowel wall thickness of sigmoid (mm), mean (SD)**	4.2 (1.7)	3.9 (1.4)
**Color doppler signal (CDS) of sigmoid (mLimberg) ≥ 2, *n* (%)**	2 (25)	4 (50)

Abbreviation: MES, Mayo endoscopic subscore.

a Stable background therapy was allowed during the study. Any dose intensification or switch of therapy was considered a study withdrawal.

### Primary efficacy outcome

**Figure 2.**
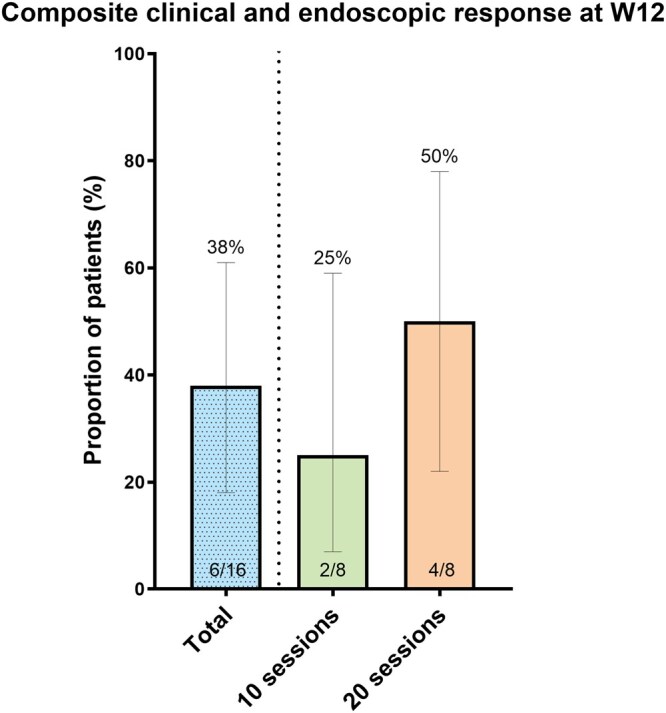
Composite clinical and endoscopic outcome at 12 weeks. Composite clinical and endoscopic outcome at 12 weeks, defined as a reduction of ≥ 3 points and 30% from baseline in the total Mayo score, a rectal bleeding subscore (RB) of 0, and a ≥ 1-point decrease in MES. All endoscopic examinations were centrally reviewed and adjudicated according to the validated VISA 2 + 1 algorithm.

At week 12, the composite clinical and endoscopic response was achieved in 6 of 16 patients (38%, 95% CI 18-61). Response rates were 25% (2/8, 95% CI 7-59) in the 10-session cohort and 50% (4/8, 95% CI 22-78) in the 20-session cohort (Figure 2). The 20-session cohort met the prespecified criteria to discontinue expansion to a 30-session cohort. Three patients discontinued the study before W12 due to lack of clinical improvement: 2 in the 10-session group and 1 in the 20-session group. Of these, 2 switched medical therapy and one underwent elective proctocolectomy.

### Secondary efficacy outcomes

At week 12, clinical remission was achieved in 3/16 patients (19%, 95% CI 7-43) and clinical response in 9/16 (56%, 95% CI 33-77; Table 2). Endoscopic improvement (MES 0-1) was observed in 3/16 (19%, 95% CI 7-43). Mean total Mayo score decreased by 4.3 points (95% CI 3.0-5.6), and UCEIS by 1.0 (95% CI 0.2-1.8) from baseline. Histologic remission (Nancy 0-1) was achieved in 3/16 (19%, 95% CI 7-43), while histologic improvement occurred in 6/16 (38%, 95% CI 18-61).

**Table 2.** Week 12 efficacy results.

	Overall *N* = 16	10 sessions HBOT *N* = 8	20 sessions HBOT *N* = 8
**Primary endpoint**			
**Composite clinical and endoscopic response**	6 (38%) [18-61]	2 (25%) [7-59]	4 (50%) [22-78]
**Key secondary endpoints**			
**Clinical remission**	3 (19%) [7-43]	0 (0%) [0-32]	3 (38%) [14-69]
**Clinical response**	9 (56) [33-77]	3 (38%) [14-69]	6 (75) [41-93]
**Endoscopic improvement (MES 0–1)**	3 (19%) [7-43]	0 (0%) [0–32]	3 (38%) [14-69]
**Change from baseline in Mayo score, mean (95% CI)**	−4.3 (−5.6 to −3.0)	−3.5 (−5.5 to −1.5)	−5.0 (−7.1 to −2.9)
**Change from baseline in UCEIS, mean (95% CI)**	−1.0 (−1.8 to −0.2)	−1.2 (−2.4 to 0.1)	−0.9 (−2.3 to 0.6)
**Histologic remission (Nancy 0–1)**	3 (19%) [7-43]	2 (25%) [7-59]	1 (13%) [2-47]
**Histologic improvement (at least 1-point improvement)**	6 (38%) [18-61]	3 (38%) [14-69]	3 (38%) [14-69]
**Symptomatic remission**	7 (44%) [20-70]	3 (38%) [14-69]	4 (50%) [22-78]

Data are presented as n (%) [95% CI] unless otherwise stated.

Biochemical markers showed FCP normalization (≤ 150 µg/g) in 7/15 (47%, 95% CI 21-73) at week 12 and 9/15 (60%, 95% CI 32-84) at week 26, and hs-CRP normalization (≤ 5.0 mg/L) in 4/5 (80%, 95% CI 28-99) and 3/5 (60%, 95% CI 15-95), respectively (Tables S1 and S2).

Patient-reported outcomes improved on the PRO-2 and IBDQ. At week 12, symptomatic remission and response were achieved in 7/16 (44%, 95% CI 20-70) and 10/16 participants (62%, 95% CI 39-82, respectively; Table S3). At week 26, this was sustained in 5/16 (31%, 95% CI 11-59) and 8/16 participants (50%, 95% CI 25-75), respectively. Health-related quality of life improved on the IBDQ, with a mean change from baseline of 18.6 points (95% CI 1.1-36.1) at the end of HBOT, sustained at week 12 (19.2, 95% CI −3.1 to 41.5) and partially attenuated by week 26 (12.2, 95% CI −6.9 to 31.4). In contrast, EQ-5D utility and VAS were stable over time. QALY accrual through week 26 was 0.42 (95% CI 0.37-0.47; Supplemental Material). Twenty-nine of 44 endoscopies (66%) required third-reader adjudication following discordance between the first 2 assessments in either MES or UCEIS, after which agreement between 2 of 3 readers was achieved for all cases.

### Contrast-enhanced ultrasound parameters

Across all analyzable ROIs (full CEUS set; 15 patients, 120 measurements), quality of fit was highest for the total bowel wall ROI (mean fit 70%, 95% CI 63-78), and all following analyses were performed on this ROI.

**Figure 3.**
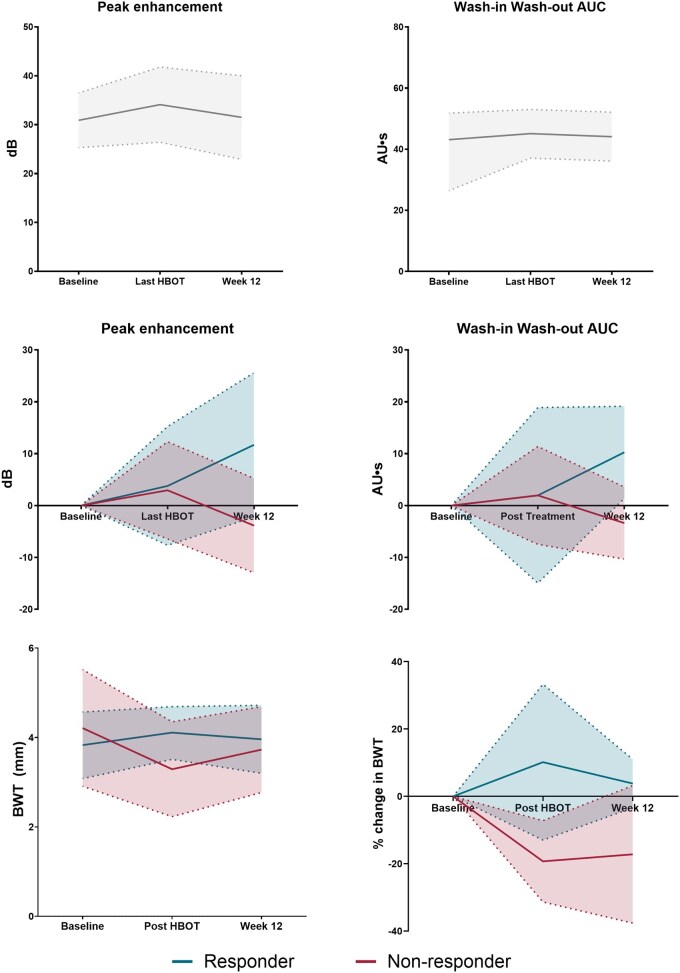
CEUS perfusion parameters and bowel wall thickness across the HBOT treatment course. Observed mean values with 95% confidence intervals (shaded areas) at baseline, the final HBOT session (10 or 20), and Week 12. (A) Peak enhancement (PE, left) and wash-in wash-out AUC (WiWoAUC, right) for the full cohort (black line) at baseline, the final HBOT session, and Week 12. (B) Change in PE and WiWoAUC stratified by Week 12 composite clinical and endoscopic response. Responders (blue) and non-responders (red) show significant different trajectories at Week 12 (PE: *P* < .03); WiWoAUC: *P* < .007). (C) absolute bowel wall thickness (BWT, left) and percentage change from baseline (right), separated by W12 composite response. Responders (blue) show a significant rise followed by attenuation at W12, whereas non-responders (red) show a significant decrease after HBOT which stabilizes to W12.

At baseline, mean peak enhancement (PE) was 30.9 dB (95% CI 25.3-26.5), and wash-in-wash-out AUC (WiWoAUC) was 43.1 AU·s (95% CI 26.4-41.8). Both perfusion parameters increased immediately, numerically after HBOT. PE rose to 34.1 dB (95% CI 26.4-41.8) and WiWoAUC to 45.1 AU·s (95% CI 37.1-53.0). By W12, values stabilized near baseline (PE 31.5 dB, 95% CI 22.9-40.0; WiWoAUC 44.1 AU·s, 95% CI 36.1-52.1; Figure 3 and Table S4).

When stratified by W12 composite clinical-endoscopic response, distinct trajectories emerged. At baseline, mean perfusion values were numerically lower in responders (PE: 26.0 dB, 95% CI 15.2-36.7; WiWoAUC: 40.0 AU·s, 95% CI 27.0-52.9) compared to non-responders (PE: 33.3 dB, 95% CI 25.9-40.8; WiWoAUC: 44.7 AU·s, 95% CI 38.1-56.7). After HBOT (T1), both groups showed a numerical rise in perfusion (ΔPE: 2.9 vs 3.8 dB; ΔWiWoAUC: 2.0 vs 2.0 AU·s, in non-responders and responders, respectively). Importantly, by W12 the trajectories diverged significantly (PE: *P* < .03; WiWoAUC: *P* < .007). Responders showed sustained increases (PE 40.3 dB [95% CI 17.5-63.1]; WiWoAUC 53.4 AU·s [36.0-70.8]), whereas non-responders showed regression or decline (PE 25.5 dB [20.1-31.0]; WiWoAUC 37.9 AU·s [31.4-44.4]) compared to their baselines.

Perfusion dynamics were comparable across the 10- and 20-session cohorts, with both showing numerical increases at T1 and stabilization at W12. No clear dose–response differences were seen, although numerically greater sustained increases were observed in the 20-session group.

Applying the quality-of-fit threshold (≥70%) restricted analyses to 11 patients (23 measurements). Patterns were directionally similar, though estimates were less precise. Across multiple perfusion−volume metrics (PE, WiAUC, WoAUC, WiWoAUC, WiPI) responders showed sustained increases, while non-responders regressed (Table S4). Other rate and timing parameters showed similar directional trends but lacked precision. Exploratory analyses suggested that any increase in PE at T1 (ΔPE > 0) identified W12 responders with 80% sensitivity (95% CI 28-99) but modest 50% specificity (95% CI 19-81), corresponding to an OR of 4.0 (95% CI 0.4-94), and an AUC of 0.66 (DeLong 95% CI 0.29-1.00; Figure S2). Changes in WiWoAUC performed similarly (AUC 0.60; 95% CI 0.21-0.99). Combining metrics offered no gain due to high collinearity (ρ  = 0.97).

### B-mode ultrasound parameters

In the sigmoid colon, mean BWT of all patients was 4.1 mm at baseline (95% CI 3.3-4.9), 3.6 mm (95% CI 2.9-4.3) at the end of HBOT and 3.8 mm (95% CI 3.3-4.4) at week 12 (Figure 3 and Table S5). At baseline, non-responders had a significantly higher BWT in the sigmoid (4.2 mm, 95% CI 2.9-5.5 vs 3.8 mm, 95% CI 3.1-4.6; *P* = .043). By the end of HBOT (T1), the BWT in responders was significantly higher compared to non-responders (4.1 mm 95% CI 3.5-4.7 vs 3.3 mm 95% CI 2.2-4.3; *P* = .012) and while in responders the BWT increased at T1 compared to baseline, in non-responders it decreased (Δ 0.3 mm, 95% CI −0.04 to 0.9 vs Δ −0.9 mm, 95% CI −1.5 to −0.3; *P* = .009). At W12, the BWT in responders was still significantly higher compared to non-responders (4.0 mm 95% CI 3.2-4.7 vs 3.7 mm 95% CI 2.8-4.7; *P* = .017) and compared to baseline the BWT remained increased in responders vs decreased in non-responders, although not significant for the absolute change in mm (Δ 0.1 mm, 95% CI −0.04 to 0.9 vs Δ −0.9 mm, 95% CI −1.5 to −0.3; *P* = .071; Table S5).

Concerning the categoric B-mode parameters, a decrease in CDS of at least ≥ 1 compared to baseline occurred in none of the responders at both T1 and W12 vs 5/10 (50%) and 6/10 (60%) of the non-responders at T1 and W12, respectively (Table S6).

### Tolerability and safety

At 26 weeks, HBOT appeared to be well tolerated. A total of 25 TEAEs occurred in 10/16 participants (62%), 80% were grade 1, and no SAEs or HBOT discontinuations occurred (Table 3 and Table S7). The most frequent TEAEs were fatigue (6/16, 38%), worsening of UC symptoms (5/16, 31%), and nasopharyngitis (2/16, 12%). TEAEs with special interest to HBOT (eg, fatigue, ear/sinus discomfort, transient myopia, and claustrophobia) were all grade 1, self-limited, and clustered during the treatment period (10 events; 5.0 EAIR per 100 HBOT sessions). During follow-up, there was no excess of HBOT-related events (0 per 100 PY). In contrast, UC related events occurred primarily during follow-up (EAIR 87.5 per 100 PY), consistent with background disease activity. There was no signal of dose-related increase in TEAE frequency between the 10- and 20-session cohorts.

**Table 3.** Overall summary of treatment-emergent adverse events (safety population, *n* = 16; 8 PY total; 240 HBOT sessions).

	Participants *n* (%)	Events (*n*)	EAIR/100 PY	EAIR/100 HBOT sessions
**TEAE**	10 (62)	25	312.5	-
**Grade ≥ 3 TEAE**	0	0	0	-
**TESAE**	0	0	0	-
**TEAE leading to treatment discontinuation**	0	0	0	0
**Treatment-related TEAEs**	8 (50)	12	150	5.0
**UC-related events**	5 (31)	7	87.5	-
**Other AEs**	5 (31)	6	75.0	-

Abbreviations: AE, adverse event; EAIR, exposure adjusted incidence rate; HBOT, hyperbaric oxygen treatment; PY, patient-years; TEAEs, treatment-emergent adverse events; TESAE, treatment-emergent severe adverse event; UC, ulcerative colitis.

## 4. Discussion

PARADOX showed that HBOT is beneficial in refractory UC patients. HBOT, proposed as a regenerative intervention through improved tissue oxygenation, angiogenesis, and immune modulation, was feasible, safe, and associated with clinical, endoscopic, and mechanistic improvements. These findings extend prior case series and retrospective observations by systematically evaluating efficacy and integrating perfusion imaging to capture mucosal responses.^7^

At week 12, composite outcome of endoscopic and clinical response was achieved in 6/16 (38%) of patients treated with 10 or 20 sessions. HBOT was well tolerated through week 26, with no TESAEs and only mild, self-limiting TEAEs. CEUS and B-mode IUS suggested a coherent mechanistic signature of HBOT. Both modalities showed modest overall changes during therapy, but significant divergence between responders and non-responders by week 12. Response was associated with an increase of several perfusion–volume metrics and preserved BWT, whereas non-responders regressed toward decreased CEUS parameters and BWT. Rate- and time-based parameters showed similar directional trends but lacked precision, supporting perfusion volume as the key mechanistic signature. Accordingly, the categorical CDS measure of macrovascular flow, decreased only in non-responders, reinforcing the microvascular changes observed by CEUS. Taken together, these findings suggest that HBOT induces early perfusion and structural improvements but only responders maintain them over time.

Our findings differ from those typically observed with biologic treatments, where successful treatment leads to reduced perfusion and BWT as inflammatory hyperemia subsides.^28–33^ In our study, however, HBOT responders showed maintained or increased B-mode and CEUS parameters, changes that would conventionally suggest ongoing inflammation. This paradoxical pattern suggests HBOT-induced neo-angiogenesis and tissue remodeling, leading to persistent edema or granulation and hyperemia, despite clinical and endoscopic improvement. These observations align with the hypothesized hyperoxia-hypoxia paradox, in which intermittent hyperoxia activates regenerative angiogenic pathways via HIF signaling upon return to normoxia.^17^^,^^18^ In contrast, non-responders demonstrated a significant reduction in BWT and CDS, suggesting distinct mechanisms underlying lack of response to HBOT. Clinically, these findings underscore that early IUS and CEUS changes during HBOT should be interpreted with caution, as IUS kinetics differs markedly from that observed under anti-inflammatory treatment alone and histological or molecular markers of angiogenesis were not assessed by this time. Taken together, our data illustrate the dual nature of elevated CEUS and CDS signals: while increased perfusion in the inflammatory setting reflects hyperemia, during HBOT, it may instead signify regenerative neovascularization and tissue repair.

The apparent dissociation between function and structure healing suggests a lag in the repair-phase, where perfusion recovery precedes structural remodeling. By week 12, this gap had narrowed, indicating that vascular improvement and tissue remodeling were beginning to converge. Although the absence of later follow-up prevents determining whether this trajectory is transient or durable. Similar perfusion-linked improvements have been described with HBOT in perianal fistulizing Crohn’s disease and radiation-induced proctitis, pointing to a recurring pattern of vascular regeneration across disease contexts.^4^^,^^34^ HBOT may further enhance concomitant anti-inflammatory therapy by improving tissue perfusion and facilitating drug delivery, potentially explaining why responders showed increases in vascular parameters yet achieved mucosal improvement. Given small sample size and baseline heterogeneity, these findings should however be interpreted with caution.

PARADOX motivates a dose-optimized randomized trial comparing 20 HBOT sessions against standard care adjunct to stable therapy with a longer follow-up for durability, safety, and cost-effectiveness. Translational analyses should test the microvascular-repair hypothesis and refine patient selection, for example by incorporating histologic or molecular markers of angiogenesis (eg, VEGF, endothelial markers, or HIF-1α). HBOT could be positioned as an adjunctive option for patients failing multiple anti-inflammatory lines, where perfusion restoration is needed to improve pharmacokinetics and consequently pharmacodynamics.

Strengths of this study include a prospective design, comprehensive multimodal assessment, central reading of endoscopic and histologic outcomes, and conservative non-responder imputation for missing endpoints. Limitations include the open-label, single-center design, the pilot nature of the study with modest sample size and absence of a placebo group. Furthermore, the sequential cohort design may have introduced baseline differences between treatment groups, which could have influenced the observed dose-ranging signals. The requirement for specialized hyperbaric chamber facilities and expertise in CEUS acquisition and analysis may limit widespread implementation. Additionally, the quality of fit was lower compared to previous studies, possibly due normalization of the BWT in the sigmoid in some patients, making measurements technically challenging.^23^^,^^28^ These findings should therefore be interpreted as hypothesis-generating and require confirmation in adequately powered randomized controlled trials.

In conclusion, the PARADOX trial demonstrates that HBOT is a well-tolerated adjunctive treatment showing preliminary clinical and endoscopic benefit in refractory UC patients. The unique vascular response identified by CEUS suggests a regenerative mechanism distinct from conventional anti-inflammatory therapies. Although practical challenges limit routine CEUS adoption, these findings strongly support further exploration of HBOT in larger controlled trials.

## Supplementary Material

jjag065_Supplementary_Data
