# Supplementary material for: Hyperbaric oxygen therapy alters bowel perfusion and improves outcomes in patients with treatment-refractory ulcerative colitis: a prospective pilot trial
Source: J Crohns Colitis. 2026 May 14;20(5):jjag065. doi: 10.1093/ecco-jcc/jjag065 (PMC13175981; doi:10.1093/ecco-jcc/jjag065)
Supplement: jjag065_Supplementary_Data [file jjag065_supplementary_data.docx]

**Supplementals**

- Supplementary Methods
- Supplementary Figure S1. CONSORT Patient flowchart
- Supplementary Table S1. Patients achieving normalization of FCP (≤150 µg/g) or hs-CRP (≤5 mg/L) during treatment and 26 weeks follow-up in patients with elevated FCP (>150 µg/g) or hs-CRP (>5 mg/L) at baseline
- Supplementary Table S2. Changes in FCP and hs-CRP during treatment and 26 weeks follow-up (n = 16)
- EQ-5D and QALY outcomes.
- Supplementary Table S3. Symptomatic remission and response during treatment and 26 weeks follow-up.
- Supplementary Table S4. CEUS parameters
- Supplementary Figure S2. Exploratory ROC/AUC plots for delta PE predicting W12 response
- Supplementary Table S5. Continuous B-mode IUS variables in the sigmoid.
- Supplementary Table S6. Categoric B-mode IUS variables in the sigmoid.
- Supplementary Table S7. Treatment-emergent adverse events (safety population, n = 16; 8 PY total; 240 HBOT sessions)

**Supplementary methods**

**Complete list of inclusion and exclusion criteria**

*Inclusion criteria*

Patients were eligible for enrollment only if they met all of the following criteria during screening:

1. Documented diagnosis of UC ≥ 4 months prior to entry into the study, confirmed with endoscopy and pathology results available in the source documents

2. Moderately to severely active UC as defined by a total MAYO score of ≥ 5 and a MAYO ES of ≥ 2 determined within 7 days of starting HBOT treatment

3. Subjects must have failed or be intolerant (discontinued the medication due to an adverse event as determined by the investigator) of the following treatments:

a. Oral corticosteroids

b. Azathioprine or 6-mercaptopurine

c. Anti-TNF therapy: infliximab, adalimumab or golimumab

d. Vedolizumab

e. Current treatment with ustekinumab (or another p19 inhibitor in a clinical trial) or small-molecule therapy (e.g., tofacitinib)

4. Current treatment with a stable dose of IL-23 or JAK inhibitors (>12 weeks of stable dose and interval of IL-23i and >6 weeks of JAKi)

5. Age 16 or older

6. Approved for compassionate use of hyperbaric oxygen therapy by the treating physician and the health insurance company

7. In the opinion of the investigator, the subject is capable of understanding and complying with protocol requirements.

8. The subject signs and dates a written, informed consent form and any required privacy authorization prior to the initiation of any study procedures.

9. Male or non-pregnant, non-lactating females. Females of child bearing potential must have a negative serum pregnancy test prior to randomization, and must use a hormonal (oral, implantable or injectable) or barrier method of birth control throughout week 26. Females unable to bear children must have documentation of such in the source records (i.e., tubal ligation, hysterectomy, or post-menopausal [defined as a minimum of one year since the last menstrual period]).

*Exclusion criteria*

Individuals were excluded from study enrollment if they met any of the following criteria:

1. Presence of indeterminate colitis, microscopic colitis, ischemic colitis, infectious colitis or clinical findings suggestive of Crohn’s disease

2. Subjects without previous treatment for UC (i.e., treatment-naïve)

3. Subjects at imminent need of surgery as judged by the treating clinician

4. Subjects with evidence of colonic adenomas or dysplasia. However, subjects with prior history of adenomatous polyps will be eligible if the polyps have been completely removed and the subjects are free of polyps at baseline

5. Subjects who have positive stool examinations for enteric pathogens (including Salmonella, Shigella, Yersinia, Campylobacter, C. difficile) detected by stool analysis within 2 weeks prior to enrollment pathogenic ova or parasites, at baseline

6. Patients with an ostomy

7. Unfit for hyperbaric oxygen therapy as assessed by the hyperbaric physician.

8. Contra-indication for endoscopy

9. Patients who received any investigational drug in the past 30 days or 5 half-lives, whichever is longer

10. A history of alcohol or illicit drug use that in the opinion of the principal investigator (PI) would interfere with study procedures

11. Patients with psychiatric problems that in the opinion of the PI would interfere with study procedures

12. Patients unable to attend all study visits

13. Patients with a history of non-compliance with clinical study protocols

**HBOT protocol**

Hyperbaric oxygen therapy (HBOT) was delivered in a multiplace hyperbaric chamber. Each session involved 100% oxygen administration at 2.4 atmospheres absolute (ATA) for 120 minutes, including 5-minute air breaks. Compression and decompression were performed at 1-2 m/min according to standard hyperbaric safety protocols. Patients were continuously monitored for symptoms of oxygen toxicity or barotrauma. Sessions compliance was documented, and sessions were considered complete if patients remained inside the chamber for the full duration.

**Endoscopic procedures**

Flexible sigmoidoscopies were performed using standard high-definition endoscopes by experienced IBD endoscopists. No sedation was used unless specifically indicated. Bowel preparation consisted of two 250 mL saline enemas administered after IUS and approximately 1 hour prior to endoscopy. Endoscopic disease activity was scored using the Mayo endoscopic subscore (MES), and mucosal biopsies were taken from the sigmoid (i.e., 15-30 cm ab ano) and proximal to the proximal margin of visible inflammation when present. Endoscopy videos were anonymized and centrally reviewed using the VISA 2+1 adjudication algorithm.

**Histological assessment**

Biopsies were fixed in formalin, embedded in paraffin, and stained with hematoxylin and eosin (H&E). Histologic inflammation was graded using the Nancy Histological Index, scored from 0 (no activity) to 4 (severely active). All samples were scored by one expert gastrointestinal pathologist, blinded to clinical data and timepoint.

**Ultrasound protocol**

Intestinal ultrasound (IUS) and CEUS were performed using an Epiq 5G ultrasound scanner (Philips, Amsterdam, The Netherlands) with a high-frequency linear probe (C5-1 convex and L12-5 linear probe) with standardized positioning. All scans were de-identified and centrally analyzed by a single trained IUS reader (M.P.), blinded to clinical status and visit timing.

Interobserver reliability was not assessed in this study, as almost perfect interobserver agreement for CEUS-derived bowel perfusion parameters using this protocol has previously been demonstrated.

**Scoring and adjudication**

Central reading and adjudication of endoscopy were performed using the validated VISA 2+1 algorithm. In brief, one independent reviewer scored each case (K.G.); in case of discordance with the local reader, a second central reader rescored the case (G.D.). Consensus was defined as agreement of 2 out of 3 scores. If consensus was not reached, an adjudication meeting was planned with the two central readers (K.G. and G.D.) to adjudicate the disagreement.

**Clinical assessments and PROs**

Secondary outcomes assessed at the end of HBOT and W12 visits included several clinical, endoscopic, histologic, biochemical, and patient-reported measures. Clinical endpoints comprised clinical remission (Mayo subscores of 0 for RB, 0 or 1 for stool frequency [SF] with a 1-point decrease from baseline, and 0 or 1 for central read endoscopy), as well as clinical response (decrease in modified Mayo score of ≥2 points and ≥35% from baseline with either a decrease of RB of ≥1 or RB of 0 or 1), symptomatic remission (SF of 0 or 1, and RB of 0) and response (at least 1-point reduction in both SF and RB, or absolute score of ≤1). Other secondary outcomes included endoscopic improvement (MES of 0 or 1), change from baseline in histological activity using the Nancy score, biochemical inflammatory parameters, and patient-reported outcome as assessed by the Inflammatory Bowel Disease Questionnaire (IBDQ), and EuroQoL Questionnaire (EQ-5D). The tolerability and safety of HBOT were evaluated based on adverse event reporting.

**Biochemical analysis**

Blood samples were collected via venipuncture and analyzed for C-reactive protein (CRP), hemoglobin, platelets, and serum albumin in the hospital’s certified clinical chemistry lab. Stool samples were self-collected within 24 hours prior to each visit and analyzed for fecal calprotectin (FCP) using a quantitative ELISA (Phadia 250 analyzer, Thermo Fischer Scientific). All assays followed manufacturer protocols and were internally validated.

**Adverse events**

Adverse events were coded according to the Medical Dictionary for Regulatory Activities (MedDRA, version 27.1), and summarized by system organ class, preferred term, severity, and relation to HBOT. A treatment-emergent AE was defined as an AE that first occurred or increased in severity following initiation of HBOT.

**Handling of Missing data**

Missing continuous data were assumed to be missing at random (MAR), based on inspection of missingness patterns and lack of correlations with baseline covariates data. Multiple imputation by chained equations (MICE) was used with 50 imputations (m=50) and 50 iterations per imputation (maxit=50). Imputation models included all outcome variables, baseline covariates, and visit indicators. Convergence was assessed via trace plots; plausibility of imputed distributions was verified against observed data using density plots and summary statistics.

**Software and tools**

Data collection utilized Castor EDC (Amsterdam, The Netherlands). All statistical analyses were conducted using R version 2024.12.0+467, with packages including lme4, lmerTest, nlme, emmeans, geepack, ez, mice, ggplot2, tableone and broom.mixed. Imaging analyses were conducted using VueBox® (Bracco Imaging) and GraphPad Prism (version 10) was used for visualizations.


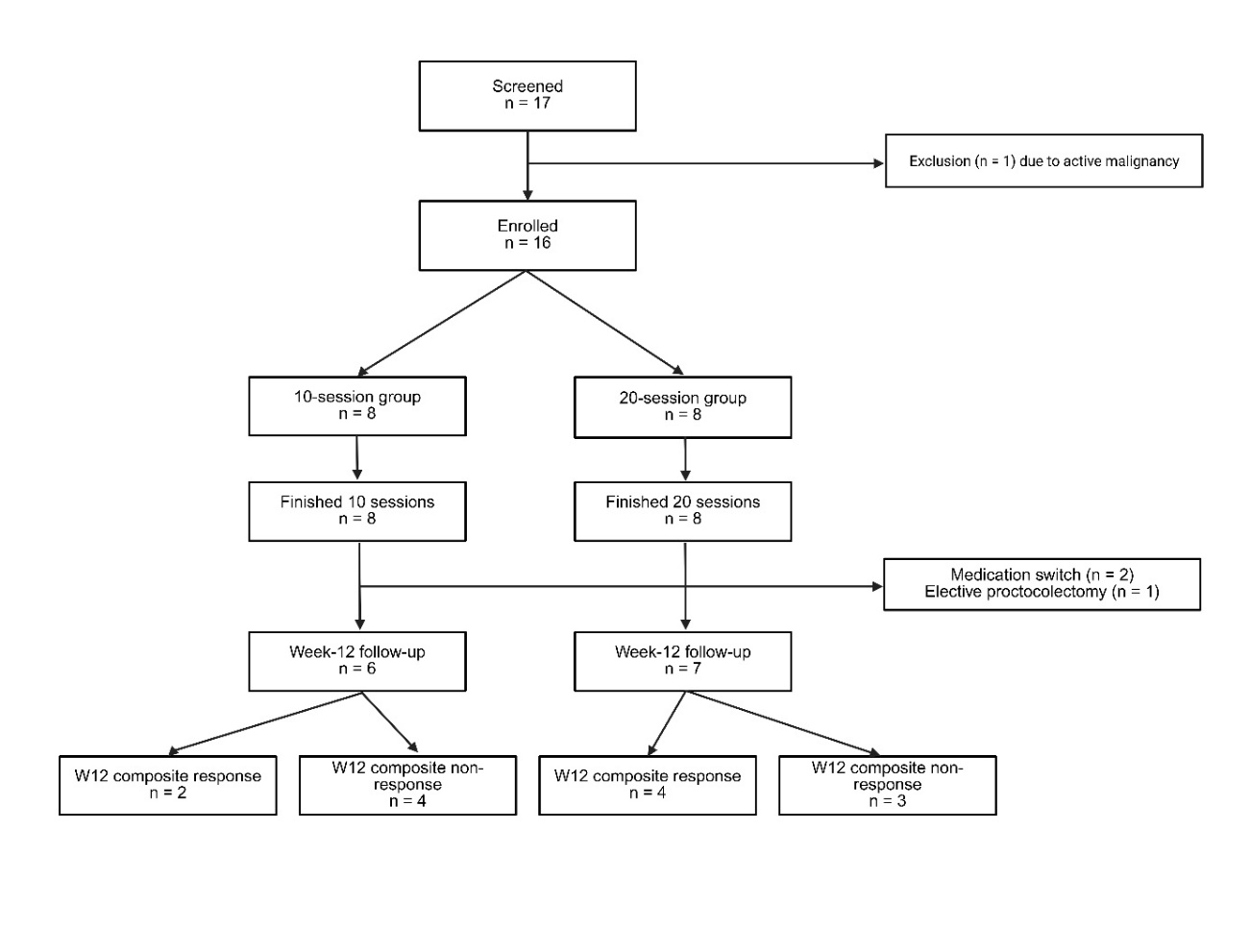
Supplementary figure S1. CONSORT Patient flowchart

Supplementary Table S1. Patients achieving normalization of FCP (≤150 µg/g) or hs-CRP (≤5 mg/L) during treatment and 26 weeks follow-up in patients with elevated FCP (>150 µg/g) or hs-CRP (>5 mg/L) at baseline

|  | FCP ≤150 µg/g, % (95% CI)  (n = 15) | hs-CRP ≤5 mg/L, % (95% CI)  (n = 5) |
| --- | --- | --- |
| Treatment phase | | |
| Day 2 | 7 (0-32) | 40 (5-85) |
| Day 6 | 20 (4-48) | 60 (15-95) |
| Day 10 | 27 (8-55) | 100 (48-100) |
| End of HBOT | 40 (16-68) | 60 (15-95) |
| Follow-up | | |
| Week 12 | 47 (21-73) | 80 (28-99) |
| Week 26 | 60 (32-84) | 50 (15-95) |

Supplementary Table S2. Changes in FCP and hs-CRP during treatment and 26 weeks follow-up (n = 16)

|  | FCP (µg/g) | hs-CRP (mg/L) |
| --- | --- | --- |
| Baseline FCP (µg/g) or hs-CRP (mg/L) | 1616.8 (612.3 – 2621.3) | 4.9 (1.1 – 8.7) |
| Treatment phase | | |
| Day 2 | 146.6 (-1084.3 to 1377.4) | -1.3 (-3.7 to 1.0) |
| Day 6 | -540.9 (-1567.5 to 485.6) | -1.2 (-3.5 to 1.0) |
| Day 10 | -830.8 (-1669.6 to 8.1) | -3.1 (-7.0 to 0.7) |
| End of HBOT | 285.4 (-1138.9 to 1709.6) | -3.6 (-2.5 to 1.3) |
| Follow-up | | |
| Week 12 | -391.9 (-1918.7 to 1134.8) | -2.1 (-5.3 to 1.2) |
| Week 26 | -948.8 (-2318.2 to 420.7) | -3.0 (-6.5 to 0.5) |

Changes from baseline, unless otherwise stated.

**EQ-5D and QALY outcomes**

Mean EQ-5D utility was 0.7 (95% CI 0.7–0.8) at baseline, 0.8 (95% CI 0.7–0.9) at the end of HBOT, and 0.7 (95% CI 0.6–0.9) at week 26. EQ-5D VAS scores followed a similar trajectory (0.6 [95% CI 0.5–0.7] at baseline, 0.7 [95% CI 0.6–0.8] at HBOT completion, 0.7 [95% CI 0.6–0.8] at week 26). QALY accrual over the treatment phase was 0.030 (95% CI 0.024–0.036) for 10 sessions and 0.056 (95% CI 0.047–0.065) for 20 sessions, corresponding to the longer treatment window. Total QALYs from baseline through week 26 were similar between cohorts (0.415 vs. 0.429).

Supplementary Table S3. Symptomatic remission and response during treatment and 26 weeks follow-up. Symptomatic remission was defined as a stool frequency subscore (SF) of 0 (or 1 with a ≥1 point decrease from baseline) and a rectal bleeding subscore (RB) of 0. Symptomatic response was defined as at least 1-point reduction in both SF and RB , or absolute score of ≤1 in combined SF and RB.

|  | Symptomatic remission  N = 16 | Symptomatic response  N = 16 |
| --- | --- | --- |
| Treatment phase | | |
| Day 2 | 13 (2-38) | 31 (11-59) |
| Day 4 | 31 (11-59) | 50 (25-75) |
| Day 6 | 31 (11-59) | 50 (25-75) |
| Day 10 | 25 (7-52) | 63 (35-85) |
| End of HBOT | 44 (20-70) | 63 (35-85) |
| Follow-up | | |
| Week 2 | 31 (11-59) | 44 (20-70) |
| Week 4 | 50 (25-75) | 63 (35-85) |
| Week 6 | 44 (20-70) | 69 (41-89) |
| Week 9 | 50 (25-75) | 81 (54-96) |
| Week 12 | 38 (15-65) | 69 (41-89) |
| Week 26 | 31 (11-59) | 50 (25-75) |

Note. Data are presented as percentage (95% CI).

Supplementary Table S4. CEUS-derived perfusion and kinetics parameters

| Parameter | Baseline mean (95% CI) | T1 mean (95% CI) | W12 mean (95% CI) | Baseline-T1 (95% CI) | *P-value* | Baseline-W12 (95% CI) | *P-value* | Baseline- W12 Responder (95% CI) | Baseline-W12 Non-responder  (95% CI) | *P-value* |
| --- | --- | --- | --- | --- | --- | --- | --- | --- | --- | --- |
| PE (dB) | 30.9  (25.3-36.5) | 34.1  (26.4-41.8) | 31.5  (22.9-40.0) | 3.2  (-3.2-9.6) | .30 | 2.4  (-5.8-10.6) | .53 | 11.7  (-2.2-25.6) | -3.9  (-13.0-5.2) | .03 |
| WiAUC (dB) | 38.3  (33.0-43.6) | 40.7  (33.1-48.3) | 39.4  (31.3-47.5) | 2.4  (-4.3-9.1) | .45 | 2.3  (-4.3-9.0) | .45 | 10.8  (1.9-19.8) | -3.3  (-10.0-3.3) | .007 |
| WoAUC (dB) | 41.4  (36.1-46.6) | 43.2  (35.4-51.1) | 42.3  (34.3-50.2) | 1.9  (-5.0-8.8) | .57 | 1.9  (-4.6-8.5) | .52 | 9.9  (0.9-18.9) | -3.4  (-10.5-3.7) | .011 |
| WiWoAUC (dB) | 43.1  (37.8-48.4) | 45.1  (37.1-53.0) | 44.1  (36.1-52.1) | 2.0  (-5.0-9.0) | .55 | 2.1  (-4.5-8.7) | .49 | 10.3  (1.4-19.2) | -3.4  (-10.3-3.6) | .009 |
| WiR (a.u./s) | 23.29  (17.15-29.43) | 27.48  (19.50-35.46) | 23.16  (14.03-32.28) | 4.19  (-2.15-10.53) | .18 | 2.18  (-7.86-12.23) | .64 | 12.01  (-8.23-32.26) | -4.37  (-16.29-7.55) | .08 |
| WoR (a.u./s) | 18.75  (12.19-25.31) | 23.25  (14.24-32.27) | 19.03  (9.55-28.52) | 4.15  (-3.31-11.61) | .25 | 2.85  (-8.30-14.00) | .58 | 13.79  (-7.83-35.42) | -4.45  (-18.00-9.10) | .08 |
| TTP (s) | 14.72  (10.45-18.99) | 14.20  (8.27-20.13) | 18.25  (8.94-27.55) | -0.52  (-5.91-4.87) | .84 | 1.59  (-9.91-13.08) | .76 | 3.99  (-37.15-45.14) | -0.02  (-7.85-7.81) | .78 |
| Rise slope (a.u./s) | 9.85  (6.77-12.93) | 8.18  (5.85-10.52) | 11.09  (6.15-16.03) | -1.67  (-4.34-1.01) | .20 | -0.21  (-7.60-7.19) | .95 | -0.32  (-24.65-24.01) | -0.13  (-7.71-7.45) | .98 |
| Fall time (s) | 23.44  (14.24-32.65) | 15.54  (11.36-19.71) | 23.28  (12.35-34.21) | -7.34  (-16.03-1.35) | .09 | -4.73  (-25.37-15.91) | .62 | -9.06  (-60.75-42.62) | -1.85  (-32.27-28.58) | .73 |
| MTT (s) | 70.91  (23.70-118.12) | 70.75  (-0.19-141.69) | 42.20  (22.48-61.93) | -0.16  (-84.57-84.25) | .99 | -33.53  (-114.33-47.28) | .37 | -3.91  (-73.98-66.17) | -53.27  (-204.01-97.46) | .46 |
| WiPI (a.u.) | 29.0  (23.4-34.6) | 32.2  (24.5-39.9) | 29.5  (21.0-38.0) | 3.2  (-3.2-9.6) | .30 | 2.3  (-5.8-10.5) | .54 | 11.6  (-2.3-25.4) | -3.9  (-12.9-5.2) | .03 |

PE, peak enhancement; WiAUC, Wash-in area under the curve; WoAUC, Wash-out area under the curve; WiWoAUC, Wash-in Wash-out area under the curve; WiR, Wash-in rate; WoR, Wash-out rate; TTP, time-to-peak; MTT, mean transit time; WiPI, Wash-in Perfusion Index

Supplementary Figure S2. Exploratory ROC/AUC plots for delta PE predicting W12 response

Supplementary Table S5. Continuous B-mode IUS variables in the sigmoid.

|  | Response (n = 6) | Non-response (n = 10) | *P-value* |
| --- | --- | --- | --- |
| Baseline (T0) | | | |
| BWT, mm (95% CI) | 3.8 (3.1 – 4.6) | 4.2 (2.9 – 5.5) | **.043** |
| Mucosa, mm (95% CI) | 0.9 (0.7 – 1.2) | 1.2 (0.7 – 1.7) | **.036** |
| Submucosa, mm (95% CI) | 1.6 (1.1 – 2.0) | 1.6 (1.1 – 2.2) | .11 |
| Last HBOT (T1) | | | |
| BWT, mm (95% CI) | 4.1 (3.5 – 4.7) | 3.3 (2.2 – 4.3) | **.012** |
| BWT change, mm (95% CI) | 0.3 (-0.4 – 0.9) | -0.9 (-1.5 to -0.3) | **.009** |
| BWT change, % (95% CI) | 10.2 (-13.0 – 33.3) | -19.3 (-31.4 to -7.2) | **.021** |
| Mucosa, mm (95% CI) | 1.0 (0.7 – 1.2) | 0.8 (0.5 – 1.1) | .26 |
| Mucosa change, mm (95% CI) | 0.1 (-0.1 – 0.2) | -0.4 (-0.7 - 0.0) | .055 |
| Mucosa change, % (95% CI) | 6.2 (-17.2 – 29.5) | -21.3 (-37.3 to -5.4) | **.037** |
| Submucosa, mm (95% CI) | 1.7 (1.0 – 2.3) | 1.2 (0.8 – 1.7) | .36 |
| Submucosa change, mm (95% CI) | 0.1 (-0.3 – 0.5) | -0.4 (-0.7 to -0.1) | **.041** |
| Submucosa change, % (95% CI) | 4.6 (-15.8 – 25.0) | -21.2 (-35.5 to -6.9) | **.027** |
| Week 12 (T2) | | | |
| BWT, mm (95% CI) | 4.0 (3.2 – 4.7) | 3.7 (2.8 – 4.7) | **.017** |
| BWT change, mm (95% CI) | 0.1 (-0.1 – 0.4) | -0.9 (-2.1 – 0.2) | .071 |
| BWT change, % (95% CI) | 3.8 (-3.4 – 11.0) | -17.2 (-37.6 – 3.2) | **.047** |
| Mucosa, mm (95% CI) | 0.8 (0.6 – 1.0) | 0.9 (0.7 – 1.2) | .26 |
| Mucosa change, mm (95% CI) | -0.1 (-0.3 – 0.1) | -0.3 (-0.8 – 0.1) | .35 |
| Mucosa change, % (95% CI) | -11.0 (-34.0 – 11.9) | -19.1 (-46.8 – 8.5) | .59 |
| Submucosa, mm (95% CI) | 1.6 (1.0 – 2.2) | 1.5 (1.0 – 2.1) | .98 |
| Submucosa change, mm (95% CI) | 0.0 (-0.3 – 0.3) | -0.3 (-1.2 – 0.5) | .17 |
| Submucosa change, % (95% CI) | -1.6 (-26.7 – 23.4) | -8.7 (-53.2 – 35.7) | .73 |

Data presented as mean and 95% confidence interval (95% CI)

Supplementary Table S6. Categoric B-mode IUS variables in the sigmoid.

| **Baseline (T0)** | **Response (n = 6)** | **Non-response (n = 10)** | *P-value* |
| --- | --- | --- | --- |
| CDS mLimberg ≥2 | 1 (17) | 5 (50) | .31 |
| Loss of haustration | 5 (83) | 6 (60) | .59 |
| Loss of stratification | 0 (0) | 1 (10) | .99 |
| Fatty wrapping | 3 (50) | 5 (50) | .99 |
| Presence of lymph nodes | 0 (0) | 0 (0) | .99 |
| **Last HBOT (T1)** |  |  |  |
| CDS mLimberg ≥2 | 2 (33) | 4 (40) | .99 |
| CDS decrease ≥1 compared to baseline | 0 (0) | 5 (50) | .093 |
| Loss of haustration | 5 (83) | 4 (40) | .15 |
| Loss of stratification | 0 (0) | 0 (0) | .99 |
| Fatty wrapping | 3 (50) | 4 (40) | .99 |
| Presence of lymph nodes | 0 (0) | 0 (0) | .99 |
| **Week 12 (T2)** |  |  |  |
| CDS mLimberg ≥2 | 2 (33) | 1 (14) | .56 |
| CDS decrease ≥1 compared to baseline | 0 (0) | 6 (86) | **.005** |
| Loss of haustration | 5 (83) | 4 (57) | .56 |
| Loss of stratification | 0 (0) | 0 (0) | .99 |
| Fatty wrapping | 2 (33) | 3 (43) | .99 |
| Presence of lymph nodes | 0 (0) | 1 (14) | .99 |

Data presented as n (percentage). CDS, color doppler signal; mLimberg, modified Limberg score;

Supplementary Table S7. Treatment-emergent adverse events (safety population, n = 16; 8 PY total; 240 HBOT sessions)

| **Adverse event category** | **Participants**  **n (%)** | **Events n** | **EAIR / 100 PY** | **EAIR / 100 HBOT sessions** |
| --- | --- | --- | --- | --- |
| Any TEAE | 10 (62) | 25 | 312.5 | - |
| Any TESAE | 0 | 0 | 0 | - |
| TEAE leading to treatment discontinuation | 0 | 0 | 0 | 0 |
| **Treatment-related TEAEs (total)** | 8 (50) | 12 | 150 | 5.0 |
| Fatigue | 6 (38) | 6 | 75 | 2.5 |
| Ear/sinus discomfort | 2 (12) | 2 | 25.0 | 0.8 |
| Myopia (transient) | 1 (6) | 1 | 12.5 | 0.4 |
| Claustrophobia | 1 (6) | 1 | 12.5 | 0.4 |
| Surgical site pain | 1 (6) | 1 | 12.5 | 0.4 |
| Hiccups | 1 (6) | 1 | 12.5 | 0.4 |
| Oxygen-toxicity symptoms | 0 | 0 | 0 | 0 |
| **UC-related events (total)** | 5 (31) | 7 | 87.5 | - |
| Worsening UC symptoms | 5 (31) | 5 | 62.5 | - |
| Rectal bleeding increase | 1 (6) | 1 | 12.5 | - |
| Abdominal pain | 1 (6) | 1 | 12.5 | - |
| **Other AEs (total)** | 5 (31) | 6 | 75.0 | - |
| Nasopharyngitis | 2 (12) | 2 | 25.0 | - |
| Headache | 1 (6) | 1 | 12.5 | - |
| Dizziness | 1 (6) | 1 | 12.5 | - |
| Menstrual irregularity | 1 (6) | 1 | 12.5 | - |
| Axillary pain | 1 (6) | 1 | 12.5 |  |

AE, adverse event; EAIR, exposure adjusted incidence rate; HBOT, hyperbaric oxygen treatment; PY, patient-years; TEAEs, treatment-emergent adverse events; TESAE, treatment-emergent severe adverse event; UC, ulcerative colitis
